# Supplementary material for: Functional tissue units in the Human Reference Atlas
Source: Nat Commun. 2025 Feb 11;16:1526. doi: 10.1038/s41467-024-54591-6 (PMC11814273; doi:10.1038/s41467-024-54591-6)
Supplement: Supplementary file 2 — Description of additional supplementary files [file 41467_2024_54591_MOESM2_ESM.pdf]

<u>Description of Additional Supplementary Files</u>

Supplemental Data 1. Vascular pathways from the heart to each of the 22 FTUs and back to the heart.
